# The VTI-VeXUS Index in Septic Shock: An Exploratory Proof-of-Concept Observational Study of a Novel Hemodynamic Parameter

**DOI:** 10.3390/jcm14165774

**Published:** 2025-08-15

**Authors:** Ross Prager, Simon Pupulin, Hawwa Chakera, Rhidita Saha, Nicolas Orozco, Jon-Emile Kenny, Philippe Rola, Michelle Yee Suet Wong, Marat Slessarev, Kimberley Lewis, Sarah Neil-Sztramko, Bram Rochwerg, John Basmaji

**Affiliations:** 1 Division of Critical Care, Western University, London, ON N6A 3K7, Canada; ross.prager01@gmail.com (R.P.); ywong252@uwo.ca (M.Y.S.W.); marat.slessarev@gmail.com (M.S.); 2 Department of Medicine, Western University, London, ON N6A 3K7l, Canada; spupulin@uwo.ca; 3 Faculty of Medicine, Western University, London, ON N6A 5C1, Canadarsaha2027@meds.uwo.ca (R.S.); 4 Centro de Investigaciones Clínicas, Fundación Valle de Lili, Cali 760001, Colombia; nicolas.orozco.e@hotmail.com; 5 Health Sciences North Research Institute, Greater Sudbury, ON P3E 2H3, Canada; jon-emile@heart-lung.org; 6 Intensive Care Unit, Santa Cabrini Hospital, CEMTL, Montréal, QC H1T 1P7, Canada; philipperola@gmail.com; 7 Department of Medicine, McMaster University, Hamilton, ON L8S 4L8, Canada; kimlewis83@gmail.com (K.L.); bram.rochwerg@gmail.com (B.R.); 8 McMaster Department of Health Research Methods, Evidence & Impact, National Collaborating Centre for Methods and Tools, Hamilton, ON L8S 4L8, Canada; neilszts@mcmaster.ca

**Keywords:** sepsis, hemodynamics, venous congestion, ultrasound, critical care

## Abstract

**Aim:** Both the arterial and venous systems independently predict mortality in septic shock, yet no bedside tools are able to integrate their assessment. Risk stratification becomes challenging when arterial parameters suggest favourable outcomes while venous parameters indicate poor prognosis, or vice versa. To address this gap, we developed the VTI-VeXUS index and conducted this proof-of-concept study to test its association with mortality. **Methods:** We conducted a prospective cohort study in two ICUs, enrolling adult patients with septic shock. We calculated the VTI-VeXUS index (VTI/[VeXUS+1]) from ultrasound measurements obtained within 24 h of ICU admission and stratified patients as having a high or low VTI-VeXUS index based on a cutoff of 11. We evaluated the primary outcome of mortality at 30 days using survival analysis. **Results:** We enrolled 62 patients. Patients with a low VTI-VeXUS index had higher rates of left ventricular dysfunction (32.3% vs. 3.2%, *p* = 0.006), right ventricular dysfunction (35.5% vs. 0.0%, *p* < 0.001), lower stroke volume (54.0 mL vs. 62.0 mL, *p* = 0.005), and increased 30-day mortality (adjusted HR: 3.86, 95% CI 1.23 to 12.14). **Conclusions:** In this exploratory proof-of-concept study, a low VTI-VeXUS index was associated with ventricular dysfunction and increased mortality. While limited by small sample size and univariate analysis, these findings suggest this novel integrated metric warrants validation in larger prospective studies.

## 1. Introduction

Despite numerous care initiatives, the morbidity and mortality from septic shock remain high [1]. Historically, early resuscitation efforts aim to improve oxygen delivery to tissues by optimizing the arterial system—augmenting preload via fluid administration, enhancing contractility with inotropes, and increasing arterial systemic vascular resistance using vasoactive medications [2,3]. These arterial parameters, particularly cardiac output and stroke volume, carry substantial prognostic value in septic shock.

However, emerging research has identified the venous system as a crucial determinant of outcomes in septic shock [4]. Specifically, venous congestion has emerged as a critical factor contributing to organ dysfunction and mortality [5,6,7]. Venous congestion develops when elevated right atrial pressure transmits retrograde to the liver, brain, kidneys, and other end organs [6,8,9,10]. Venous congestion precipitates tissue afterload; even modestly elevated pressures (e.g., right atrial pressure of 12mmHg) impair tissue perfusion [11]. Tools like the Venous Excess Ultrasound Score (VeXUS) now allow bedside quantification of this venous congestion and are independently associated with adverse patient outcomes.

Although both arterial and venous parameters independently predict mortality in septic shock, their combined prognostic significance remains unexplored. Clinicians particularly need guidance when these systems diverge, such as when one appears reassuring while the other signals deterioration. Does a patient with preserved cardiac output but severe venous congestion have a better prognosis than one with low cardiac output but no venous congestion? When both systems are compromised, is mortality risk additive or synergistic? Without understanding these relationships, clinicians cannot accurately assess risk or prioritize interventions. This knowledge gap is particularly relevant during the transition from emergency department stabilization to ICU care, where initial management focuses on hemodynamic optimization, but risk stratification remains challenging [12].

Similarly to how the shock index (a simple ratio of heart rate to systolic blood pressure) enhances early detection of hemodynamic compromise by combining heart rate and blood pressure, integrating arterial and venous ultrasound parameters may identify high-risk states which are not apparent when considering each parameter separately. Building on our prior work, we propose the novel VTI-VeXUS index—the first metric to combine the left ventricular outflow tract (LVOT) velocity time integral (VTI) (a surrogate for the arterial system) with Venous Excess Ultrasound Score (VeXUS; a surrogate for the venous system). Like the shock index, this value could facilitate rapid bedside risk stratification when individual parameters portend conflicting prognostic outlooks.

This exploratory study represents the initial proof of concept for the VTI-VeXUS index in septic shock. By combining the left ventricular outflow tract VTI with VeXUS measurements, we seek to establish whether this integrated metric provides prognostic value. If lower VTI-VeXUS indexes are associated with increased mortality, this would justify larger studies to precisely quantify the index’s prognostic accuracy and establish optimal thresholds for clinical decision-making.

## 2. Materials and Methods

### 2.1. Ethics Approval

This study was approved by the Western University (London, ON, Canada) Research Ethics Board (WREM Approval #120202). We obtained consent from all patients or their substitute decision-makers using a deferred consent model. This enabled the inclusion of critically ill patients who were unable to provide consent at the time of enrollment.

### 2.2. Study Reporting

The study was designed and reported following the Strengthening the Reporting of Observational Studies in Epidemiology (STROBE) guidelines [13].

### 2.3. Study Design and Setting

We conducted a prospective, multi-centre observational cohort study in two tertiary intensive care units (ICUs) in London, Ontario, Canada. These ICUs care for medical–surgical, transplant, trauma, and neurological patients, and together provide 64 critical care beds. A dedicated critical care ultrasound service performed focused echocardiograms and VeXUS measurements.

### 2.4. Participants

We enrolled adult patients (≥18 years old) diagnosed with septic shock based on Sepsis-3 criteria within 12 h of ICU admission. Additional inclusion criteria included at least one feature of end-organ dysfunction: serum lactate ≥2.0 mmol/L, acute kidney injury (AKI) of at least Acute Kidney Injury Network Stage I, Glasgow Coma Scale (GCS) <13, or the need for mechanical ventilation. We excluded patients with limitations on life support interventions, those who had undergone liver transplantation, or patients with pre-existing end-stage renal disease requiring dialysis.

### 2.5. Ultrasound Measurements

Trained operators performed ultrasound measurements: those with fellowship training in critical care ultrasound who completed specific training in VeXUS. This training consisted of a one-hour didactic session addressing the use of VeXUS, followed by direct supervision for the first three scans to ensure competency. Study investigators with expertise in VeXUS and echocardiography reviewed all ultrasound measurements and echocardiographic images in real-time to ensure accuracy and consistency. When a technique required refinement, investigators provided immediate feedback and operators repeated measurements until they achieved adequate image quality and proper technique. Ultrasound assessments included evaluations of the inferior vena cava, hepatic, portal, and intra-renal veins within 24 h of ICU admission. The VeXUS protocol is highly reproducible among ultrasound operators. For example, the interclass correlation coefficient for portal vein pulsatility was greater than 0.9, while the Cohen’s Kappa agreement for determining the flow pattern of the intrarenal vein was greater than 0.8 [14]. With respect to the interpretation of the VeXUS scan, the inter-rater agreement in was 0.95 (95% CI 0.9 to 1.0) [8].

Possible VeXUS scores range from 0 to 3. Scores of 0 or 1 represented ‘no venous congestion’ and scores of 2 or 3 represented moderate to severe ‘venous congestion’ [7]. We assessed left ventricular (LV) and right ventricular (RV) function and dichotomized findings into normal or abnormal. We defined LV dysfunction as an ejection fraction of less than 50% qualitatively. Similarly, we defined RV dysfunction qualitatively and based on semi-quantitative measurements like the tricuspid valve s’ (TV S’) velocity or tricuspid annual plane systolic excursion (TAPSE). We assessed the severity of tricuspid valve regurgitation using colour Doppler, with moderate and severe tricuspid regurgitation grouped for analyses. We estimated the stroke volume using the LVOT VTI and the LVOT diameter.

We calculated the VTI-VeXUS index by dividing the LVOT VTI by the VeXUS score + 1 (VTI/[VeXUS+1]). We added 1 to the VeXUS score before calculating the index to prevent undefined values resulting from division by zero.

### 2.6. Data Collection

Trained research assistants collected data from both paper and electronic health records. We entered information describing demographics, clinical characteristics, ultrasound measurements, and patient outcomes into a Redcap (Research Electronic Data Capture) database. To ensure accuracy, outcome data were reviewed in duplicate by a study investigator, blinded to the patient’s venous congestion status and echocardiographic data.

### 2.7. Sample Size Justification

We powered this proof-of-concept study to detect an unadjusted hazard ratio of 3.5 for 30-day mortality between patients with a high versus a low VTI-VeXUS index. Although this represents a large effect size, three considerations justified this approach. First, unadjusted estimates typically attenuate after covariate adjustment; starting with a sizeable effect maximizes the likelihood that a clinically relevant signal will remain once we undertake an adjusted analysis in the next phase of this research program. Second, the prognostic strength of related physiological constructs is already demonstrated in this range: moderate to severe venous congestion (VeXUS grade 2–3) [15,16] and left ventricular dysfunction each independently carry a hazard ratio of 3 to 4 for mortality [17,18,19]. For our new index to add clinical value, it must at minimum rival or exceed these established benchmarks. Based on a two-sided alpha of 0.05, a beta coefficient of 0.20, a hazard ratio of 3.5, and a mortality rate of 30–35% at 30 days, we require at least 60 participants.

### 2.8. Data Analysis

We analyzed data using R (version 4.4.1) in order to stratify patients into low and high VTI-VeXUS indexes. We dichotomized patients using a VTI-VeXUS index cutoff of 11, based on a physiologic framework inspired by Diamond-Forrester hemodynamic profiling [20]. This framework categorizes patients by arterial flow (normal VTI: >20–22 cm) and venous congestion (moderate to severe congestion: VeXUS score 2–3; no to mild venous congestion: VeXUS: scores 0–1). A normal profile, with a definitively normal stroke volume (VTI 22 or higher) with no or mild venous congestion (VeXUS 0 or 1), yields an index of at least 11 (VTI of 22 divided by VeXUS ≤ 1 plus 1). We selected 11 as the cutoff, representing a value that corresponds with normal hemodynamic coupling as well as the median value in the data.

We then compared baseline characteristics, demographics, comorbidities, clinical data, echocardiographic features, and outcomes. We summarized categorical variables as counts, percentages, and continuous variables as medians with interquartile range (IQR), given that the data were not normally distributed. We used the Wilcoxon rank sum test to compare differences in continuous data, and the Chi-square or Fisher’s exact test to compare differences in categorical data. We performed Cox proportional hazards regression for the primary outcome of 30-day mortality. We first conducted univariate analysis followed by multivariable analysis to address potential confounding. We selected age and MODS as covariates based on their established associations with mortality in patients with septic shock. We used univariate linear and logistic regression models to determine the association between VTI-VeXUS index and secondary outcomes, including major adverse kidney events (MAKE) at 30 days (a composite of persistent creatinine elevation of >200% baseline, dialysis, or death at 30 days), renal replacement therapy (RRT) at 30 days, duration of vasoactive agents, duration of mechanical ventilation, and ICU length of stay. We generated survival curves using the Kaplan–Meier method. All *p*-values were two-sided, with statistical significance defined as *p* < 0.05.

## 3. Results

We screened 350 patients for eligibility between 1 January 2022 and 1 January 2023. Of those, we included 62 patients in the study.

### 3.1. Baseline Characteristics

The median age was 64.0 years (IQR 56.0 to 73.0), and 37 (59.7%) patients in the cohort were female. The median VTI-VeXUS index was 11.4 (IQR 7.6 to 17.07). Between the high- and low-VTI-VeXUS-index groups, we found no significant differences in age, sex, severity of illness score defined by the multiorgan dysfunction score (MODS), or comorbidities. The primary source of sepsis was pulmonary in 83.9% of patients, with a higher frequency in the high-VTI-VeXUS group (96.8% vs. 71.0%, *p* = 0.016). Intra-abdominal sources of sepsis were more common in the low-VTI-VeXUS group compared to patients in the high-VTI-VeXUS group (29.0% vs. 3.2%, *p* = 0.016). Table 1 summarizes patient demographics.

Between the high- and low-VTI-VeXUS groups, we also found no differences in mean arterial pressure admission, heart rate, or vasopressor doses at the time of ICU admission. Moreover, we found no differences in the use of inotropes, positive pressure ventilation, fluid balance pre-VeXUS, or laboratory data at the time of ICU admission (lactate, creatinine, white blood cells, platelets, or hemoglobin). Table 2 summarizes clinical variables and laboratory data.

### 3.2. Echocardiographic Data

Patients with a low VTI-VeXUS index had higher rates of RV dysfunction (35.5% vs. 0%, *p* < 0.001) and LV dysfunction (32.3% vs. 3.2%, *p* = 0.006). Patients with a low VTI-VeXUS index also had reduced stroke volumes (54.0 mL vs. 62.0 mL, *p* = 0.005). reduced VTI (16.0 cm vs. 18.2 cm, *p* = 0.003), and reduced TV S’ (12 vs. 14.2 cm/second, *p* = 0.027). However, we found no differences in cardiac output, TAPSE, or frequency of moderate to severe TR between the two groups (see Table 3).

### 3.3. Patient Outcomes

**Figure 1:**
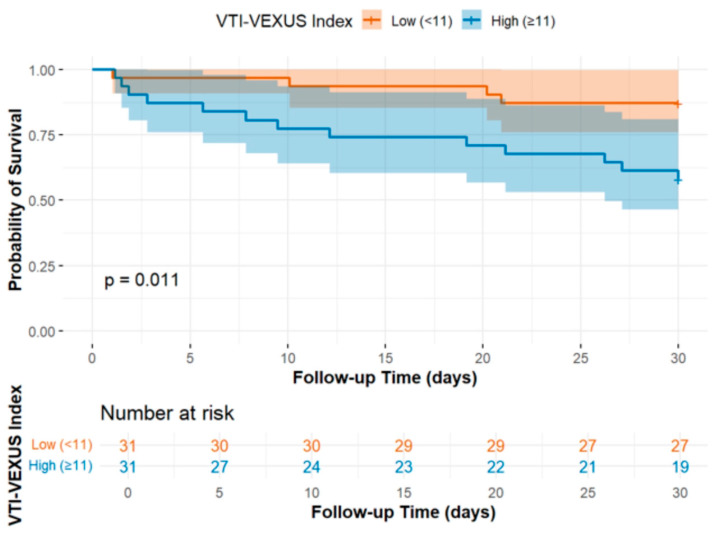
Mortality over 30 days for patients with a high or low VTI-VeXUS index.

Patients with a low VTI-VeXUS index demonstrated an increased risk of death at 30 days (HR 3.83, 95% CI: 1.25 to 11.78) compared to patients with a high index (Table 4). Patients with a high VTI-VeXUS index also demonstrated increased survival compared to patients with a low VTI-VeXUS index (*p* = 0.011) (Figure 1). We found no important differences between high- and low-VTI-VeXUS-index groups for MAKE-30 (OR = −0.06, 95% CI: −0.15 to 0.03), in terms of initiation of new renal replacement therapy (OR = 0.02, 95% CI: −0.10 to 0.13), duration of vasopressor use (β = −0.03, 95% CI: −0.21 to 0.14), duration of mechanical ventilation (β = −0.1, 95% CI: −0.41 to 0.21), or ICU length of stay (β = −0.25, 95% CI: −0.75 to 0.7) (Table 4).

## 4. Discussion

In this exploratory proof-of-concept study, we present the first evaluation of the novel VTI-VeXUS index in critically ill patients. Patients with a low VTI-VeXUS index exhibited increased 30-day mortality. While we must interpret these unadjusted results with caution, the magnitude of this effect suggests potential clinical significance that merits further investigation. Despite methodological limitations, these findings indicate that combining arterial flow with venous congestion measurements may capture important physiological derangements in septic shock. Our results warrant validation through larger prospective studies with multivariable adjustment.

In septic shock, arterial parameters such as cardiac output have long been recognized as important predictors of mortality. Consequently, resuscitation paradigms have traditionally focused on optimizing forward flow through the administration of fluids and vasoactive medications. However, recent evidence has established the venous system as an equally important determinant of outcomes. Venous congestion, now quantifiable through tools like the VeXUS score, independently predicts organ failure and mortality. Despite growing recognition that both systems influence outcomes, current practice evaluates them separately. This approach creates clinical uncertainty when arterial and venous parameters portend divergent prognoses. For instance, when preserved cardiac output suggests a favourable outlook while severe venous congestion indicates a high mortality risk. The lack of integrated hemodynamic parameters leaves clinicians without tools to assess the combined impact of arterial and venous dysfunction. Based on our preliminary findings, the VTI-VeXUS index addresses this gap by combining these complementary measurements into a single metric, capturing hemodynamic information that neither parameter alone can provide.

In septic shock, a low VTI-VeXUS index likely reflects ventricular-vascular uncoupling, where forward flow is insufficient relative to the degree of venous congestion. Even with relatively preserved stroke volumes, elevated venous pressures create tissue afterload and impair microcirculatory flow, reducing effective perfusion gradients across vital organs. This pathophysiological state, where arterial flow cannot overcome the deleterious effects of venous congestion, may explain why patients with low VTI-VeXUS values experience higher rates of mortality.

While our study provides the first evidence for the VTI-VeXUS index in septic shock, other integrated hemodynamic indices have demonstrated similar conceptual value in different populations. For example, the Pulmonary Arterial Pressure Index (PAPi) combines measurements of right ventricular stroke volume surrogate and central venous pressure to identify patients at risk of adverse outcomes. In patients with heart failure, a low PAPi is associated with an increased risk of mortality and the need for mechanical circulatory support [21,22,23]. However, the PAPi evaluates only the right ventricular function and necessitates invasive pulmonary artery catheterization, limiting its broader use in critical care. The VTI-VeXUS index shares the PAPi’s biological line of integrating arterial and venous hemodynamics but is assessed using non-invasive point-of-care ultrasound.

In our group’s previous work, we outlined a conceptual framework for integrated assessment of arterial and venous systems [20,24]. The present study provides empirical support for this approach, demonstrating that the VTI-VeXUS index captures stroke volume and venous congestion in a unified physiological profile. This index offers several distinct advantages: bedside acquisition using widely available ultrasound technology, repeatability for serial monitoring, and applicability across diverse clinical settings. These features enable resuscitations to track hemodynamic trajectories and therapeutic responses over time. Moreover, by shifting from an isolated assessment of individual systems toward integrated hemodynamic evaluation, the index represents a conceptual advancement in septic shock management. Beyond hemodynamic assessment, the VTI-VeXUS index may enable clinicians to stratify risk, support triage and admission decisions, and tailor monitoring intensity for patients with septic shock. Its non-invasive nature and ease of use could facilitate broader adoption across various clinical settings. Future studies should prospectively validate the VTI-VeXUS index, clarify optimal thresholds, and determine whether its use improves patient outcomes and guides individualized therapy.

The VTI-VeXUS index may help guide therapeutic decisions in complex hemodynamic scenarios. For patients with a low index, clinicians face two potential strategies: improving forward flow and/or reducing venous congestion. When high vasopressor requirements preclude aggressive decongestion, augmenting VTI through inotropic support may be the safer initial approach. Conversely, in patients with adequate blood pressure on minimal vasopressors, gentle diuresis or ultrafiltration might address venous congestion components. Serial VTI-VeXUS measurements could track response to either strategy, with an improving index portending benefit regardless of the approach chosen. As a result, the VTI-VeXUS index may serve as a flexible and objective marker to guide and monitor therapeutic choices.

Despite its promise, the VTI-VeXUS index has important limitations. Like the shock index or the PAPi, it cannot identify the underlying cause of shock or venous congestion. Conditions such as RV failure, severe tricuspid regurgitation, pericardial effusion, pulmonary hypertension, or biventricular failure can all produce low cardiac output with venous congestion. Clinicians should interpret a low VTI-VeXUS index as an early warning sign or as a signal to conduct further diagnostic evaluation, not as an instruction to initiate specific therapies such as decongestion or inotrope administration.

This study has several strengths. This study represents the first to empirically evaluate the VTI-VeXUS index—a novel, non-invasive index integrating arterial and venous physiology in septic shock. We leveraged prospectively collected data, applied standardized ultrasound protocols, and ensured blinded outcome assessment to minimize bias. The VTI-VeXUS index can be obtained rapidly at the bedside using widely available ultrasound technology, supporting scalability and implementation in diverse settings.

This study has several limitations. The small sample size yielded imprecision in the confidence intervals (CI) around our point-estimate. Moreover, we enrolled patients at two hospitals which may limit generalizability. Importantly, our results are unadjusted and remain exploratory as a proof of concept for this novel index; therefore, our effect size may be overestimated. However, this study was designed as a proof of concept to determine whether a signal exists that justifies investment in future prospective studies to validate this metric.

## 5. Conclusions

This exploratory study provides preliminary evidence that the VTI-VeXUS index—a novel integration of arterial flow and venous congestion measurements—may identify patients at increased risk of mortality due to septic shock. Although limited by sample size and univariate analysis, the magnitude of the relative hazard of death at 30 days suggests that this non-invasive metric warrants further investigation. We require future prospective studies with larger cohorts and multivariable adjustment to validate these findings, establish optimal thresholds, and determine whether the VTI-VeXUS index can guide clinical decision-making in septic shock.

## Figures and Tables

**Table 1:** Patient demographics for low- and high-VTI-VeXUS index.

Demographic Variables	Overall, N = 62	High VTI-VeXUS Index N = 31	Low VTI-VeXUS Index N = 31	*p*-Value
Age (Q1, Q3)	64.0 (56.0, 73.0)	64.0 (56.0, 73.0)	63.0 (54.0, 72.5)	0.9
Male n (%)	25 (40.3%)	11 (35.5%)	14 (45.2%)	0.6
Body Mass Index (kg/m^2^)	26.7 (21.2, 33.1)	27.0 (23.6, 34.1)	25.6 (20.2, 31.9)	0.4
MODS (IQR)	4.0 (3.0, 6.0)	4.0 (2.0, 6.0)	5.0 (3.3, 8.0)	0.14
**Comorbidities, n (%)**				
CHF	3 (4.8%)	2 (6.5%)	1 (3.2%)	>0.9
CAD	7 (11.3%)	1 (3.2%)	6 (19.4%)	0.1
CKD	3 (4.8%)	1 (3.2%)	2 (6.5%)	>0.9
Stroke	3 (4.8%)	3 (9.7%)	0 (0.0%)	0.2
Cirrhosis	1 (1.6%)	0 (0.0%)	1 (3.2%)	>0.9
COPD	8 (12.9%)	5 (16.1%)	3 (9.7%)	0.7
Atrial Fibrillation	7 (11.3%)	2 (6.5%)	5 (16.1%)	0.4
Diabetes	14 (22.6%)	8 (25.8%)	6 (19.4%)	0.8
**Source of Sepsis**				
Pulmonary	52 (83.9%)	30 (96.8%)	22 (71.0%)	0.016
Intraabdominal	10 (16.1%)	1 (3.2%)	9 (29.0%)	0.016

Abbreviations: *MODS*: Multi-organ dysfunction score; *CKD*: Chronic Kidney Disease; *COPD*: Chronic Obstructive Pulmonary Disease; *CHF*: Congestive Heart Failure; *CAD*: Coronary Artery Disease; *Afib*: Atrial fibrillation.

**Table 2:** Clinical variables for patients with high- and low-VTI-VeXUS index.

Clinical Variables	Overall, N = 62	High VTI-VeXUS Index, N = 31	Low VTI-VeXUS Index, N = 31	*p*-Value
MAP at time of admission, mmHg, median (IQR)	70.5 (64.3, 84.0)	70.0 (65.0, 87.5)	71.0 (64.0, 80.5)	0.6
HR at time of admission, bpm, median (IQR)	93.0 (82.3, 111.5)	90.0 (81.5, 104.5)	99.0 (84.0, 110.0)	0.3
Highest vasopressor dose (NE equivalents) on day 1 (mcg)	0.30 (0.11, 0.40)	0.2 (0.13, 0.30)	0.30 (0.11, 0.40)	0.6
Use of inotropes on day 1, n (%)	10 (16.1%)	3 (9.7%)	7 (22.6%)	0.3
Positive Pressure Ventilation at time of POCUS day 1, n (%)	40 (64.5%)	16 (51.6%)	24 (77.4%)	0.063
Fluid balance before day 1 VEXUS mL, median (IQR)	1112.0 (425.0, 3434.0)	1000.0 (225.0, 3280.0)	1533.0 (628.5, 3264.5)	0.3
**Laboratory Data**				
Lactate, mmoL/L, median (IQR)	2.2 (1.6, 4.0)	1.9 (1.3, 3.3)	3.1 (1.9, 4.1)	0.075
Baseline Creatinine, μmol/L, median (IQR)	93.0 (65.5, 107.0)	94.0 (71.5, 107.0)	92.0 (61.0, 107.0)	0.5
WBC × 10^9^/L, median (IQR)	17.0 (10.0, 26.0)	14.0 (9.0, 23.5)	17.5 (11.3, 27.5)	0.4
Hemoglobin, g/dL, median (IQR)	103.5 (86.8, 123.8)	108.0 (88.5, 123.5)	103.0 (87.5, 124.5)	0.7
Platelets × 10^9^/L, median (IQR)	214.5 (137.0, 273.5)	217.0 (120.0, 277.0)	212.0 (144.0, 265.5)	0.6

Abbreviations: MAP: Mean Arterial Pressure; HR: Heart Rate; NE: Norepinephrine equivalents; POCUS: Point of care ultrasound.

**Table 3:** Echocardiographic data for patients with high and low VTI-VeXUS.

Echocardiography Variables	Overall, N = 62	High VTI-VeXUS Index, N = 31	Low VTI-VeXUS Index, N = 31	*p*-Value
LV dysfunction, n (%)	11 (17.7%)	1 (3.2%)	10 (32.3%)	0.006
RV dysfunction, n (%)	11 (17.7%)	0 (0.0%)	11 (35.5%)	<0.001
RV dilation, n (%)	19 (30.6%)	4 (12.9%)	15 (48.4%)	0.006
Moderate or Severe TR, n (%)	8 (13.1%)	2 (6.7%)	6 (19.4%)	0.3
VTI (cm), median (IQR)	17.1 (15.3, 19.8)	18.2 (16.7, 21.1)	16.0 (13.9, 18.9)	0.003
Stroke volume, mL, median (IQR)	58.0 (50.4, 67.0)	62.0 (55.0, 70.0)	54.0 (47.0, 65.2)	0.005
Cardiac Output L/min, median (IQR)	5.3 (4.1, 6.0)	5.4 (4.7, 6.4)	5.1 (3.5, 5.6)	0.088
TAPSE, mm, median (IQR)	21.8 (17.9, 24.8)	21.7 (18.8, 24.4)	22.0 (16.6, 24.9)	>0.9
TV S’, cm/s, median (IQR)	13.4 (10.8, 15.1)	14.2 (12.4, 15.7)	12.0 (8.9, 14.5)	0.027

Abbreviations: LV: Left Ventricle; RV: Right Ventricle; TR: tricuspid regurgitation; VTI: velocity time integral; TAPSE: Tricuspid Annular Plane Systolic Excursion; TV S’: Tricuspid Annular Systolic Velocity.

**Table 4:** Outcomes for patients with high and low VTI-VeXUS.

Outcome	Estimate of Effect (95% CI)	*p*-Value
**Primary Outcome**		
30-Day Mortality (univariate)		
Low VTI-VeXUS Index (<11)	HR 3.83 (95% CI 1.25 to 11.78)	**0.018**
30-Day Mortality (multivariable)		
Low VTI-VeXUS Index (<11)	HR 3.86 (95% CI 1.23 to 12.14)	**0.021**
Age	HR 0.99 (95% CI 0.96 to 1.03)	0.745
MODS	HR 1.03 (95% CI 0.88 to 1.20)	0.717
**Secondary Outcomes**		
MAKE-30	OR −0.06 (95% CI −0.15 to 0.03)	0.188
New renal replacement therapy start at 30 days	OR 0.02 (95% CI −0.10 to 0.13)	0.761
Duration of vasoactive medications, days	β −0.03 (95% CI −0.21 to 0.14)	0.703
Duration of mechanical ventilation, days	β −0.1 (−0.41, 0.21)	0.532
ICU length of stay, days	β −0.25 (−0.7, 0.70)	0.946

Abbreviations: β: Beta coefficient; CI: Confidence Interval; HR: Hazard Ratio; ICU: Intensive Care Unit; MAKE-30: Major Adverse Kidney Events at 30 days; MODS: Multiorgan Dysfunction Score OR: Odds Ratio.

## Data Availability

Data is available upon request.
